# Clinical Significance of Glucose to Lymphocyte Ratio (GLR) as a Prognostic Marker for Patients With Pancreatic Cancer

**DOI:** 10.3389/fonc.2020.520330

**Published:** 2020-09-30

**Authors:** Ailing Zhong, Chien-shan Cheng, Jinyan Kai, Renquan Lu, Lin Guo

**Affiliations:** ^1^Department of Clinical Laboratory, Shanghai Cancer Center, Fudan University, Shanghai, China; ^2^Department of Oncology, Shanghai Medical College, Fudan University, Shanghai, China; ^3^Department of Integrative Oncology, Shanghai Cancer Center, Fudan University, Shanghai, China

**Keywords:** pancreatic cancer, prognosis, glucose to lymphocyte ratio, tumor biomarker, nomogram

## Abstract

Glucose metabolism and systemic inflammation have been associated with cancer aggressiveness and patient prognosis in various malignancies. This study aimed to evaluate the prognostic significance of pretreatment GLR(glucose to lymphocyte ratio) and systemic immune inflammation in patients with pancreatic cancer. We studied 360 patients with pathologically diagnosed pancreatic adenocarcinoma that was clinically unresectable. Baseline clinicopathological characteristics and laboratory investigations including fasting blood glucose, platelet count, lymphocyte count, neutrophil count, carcinoembryonic antigen (CEA), carbohydrate antigen 19-9 (CA199), and follow-up data were collected for further analysis. The patients were randomly divided into a training cohort (*n* = 238) and a validation cohort (*n* = 122). Univariate and multivariate Cox proportional hazard regression analyses were performed to identify the prognostic value of GLR, systemic immune-inflammation markers, and tumor biomarkers. A nomogram model was developed based on the identified prognostic factors, and we used the C-index to evaluate the accuracy of the Cox regression model prediction. Multivariate analysis revealed that GLR [hazard ratio (HR): 2.597; 95% confidence interval (CI): 1.728–3.904)] and CA199 (HR: 2.484; 95% CI: 1.295–4.765) are independent predictors of poor overall survival in the training cohort and were incorporated into the nomogram for OS as independent factors. Moreover, the C-index analyses demonstrated that the C-indexes in the training cohort and the validation cohort were 0.674 and 0.671, respectively. The nomogram model predicts overall survival relatively accurately. We found that the baseline GLR is an independent prognostic factor for patients with pancreatic cancer, and the proposed nomogram can be used as an effective tool for predicting the outcomes of prognosis of patients with pancreatic cancer.

## Introduction

Pancreatic cancer is a highly lethal malignant tumor with a 5-year survival rate of <5% (1). Ranked as the fourth leading cause of cancer-related mortality globally, the poor prognosis of pancreatic cancer may be attributed to its invasive phenotype, resistance to treatment, and the lack of early diagnostic methods (2). Surgical resection is the only curative treatment option for pancreatic cancer; however, more than 80% of patients are diagnosed at advanced and inoperable stages. For patients with advanced disease and distant metastasis, despite efforts being made for the development of novel therapeutic strategies, the overall survival has not improved substantially over the last decade (3). Other than systemic gemcitabine administration, other regimens such as FOLFIRINOX (leucovorin, fluorouracil, irinotecan, and oxaliplatin), gemcitabine plus nab-paclitaxel, and gemcitabine plus erlotinib are associated with significantly increased toxicity compared with gemcitabine monotherapy and they only provide limited survival benefits with a median survival of ~5 to 6 months, and a 5-year survival rate of ~ 8% (4–7). Therefore, it is particularly important to identify molecular markers that can be used to predict patient outcomes and for tailoring optimal treatment strategies to individual patients with inoperable pancreatic cancer.

Prognostic evaluation allows for tailoring personalized treatment for patients with inoperable pancreatic cancer to maximize short- and long- term oncological outcomes. Currently, numerous prognostic factors have been developed. Commonly applied indicators such as histological grade, lymph node involvement or distant metastasis, vascular and perivascular infiltration, and CA199 have been used to predict individual patient outcomes. However, the clinical benefits have so far been limited due to the diversification as the disease progresses and the limited number of available serum biomarkers. Therefore, the need for a more accurate and comprehensive assessment system with improved sensitivity and specificity for prognostication persists and is of high clinical value.

In the last decade, increasing research attention has become focused on the role of cancer-related inflammation in disease progression, the tumor microenvironment, metastasis, and the response to systemic therapies. Furthermore, increasing numbers of studies have focused on the clinical interpretation of inflammation factors and their prognostic value (8–10). In addition, diabetes and elevated fasting blood glucose levels have been associated with an increased risk of multiple neoplasms of the gastrointestinal tract (11), and elevated fasting blood glucose levels may affect the clinical outcomes and overall survival of cancer patients (12–14). However, to the best of our knowledge, no studies regarding fasting blood glucose and systemic inflammatory response markers in patients with pancreatic cancer have been published so far.

This retrospective study evaluated the prognostic role of fasting blood glucose levels, systemic inflammatory response markers, and tumor biomarkers in pretreatment non-resectable pancreatic cancer patients to develop a novel prognostic model. We identified a novel factor with good sensitivity and specificity, the ratio of glucose to lymphocytes (GLR), a parameter of both glucose metabolism (associated with cancer invasiveness), and systemic immune status in patients with non-resectable pancreatic cancer. A nomogram model was established to provide an accurate multivariate clinical prognostic evaluation system for patients with non-resectable pancreatic cancer. This finding provides a new basis and a reference for the clinical treatment of pancreatic cancer.

## Materials and Methods

### Patients

This study was approved by the Ethics Committee of Fudan University Shanghai Cancer Center. Written informed of consent was obtained from each participant in accordance with institutional guidelines. All procedures were performed according to the ethical standards of the 1964 Helsinki Declaration and its later amendments or comparable ethical standards.

**Figure 1:**
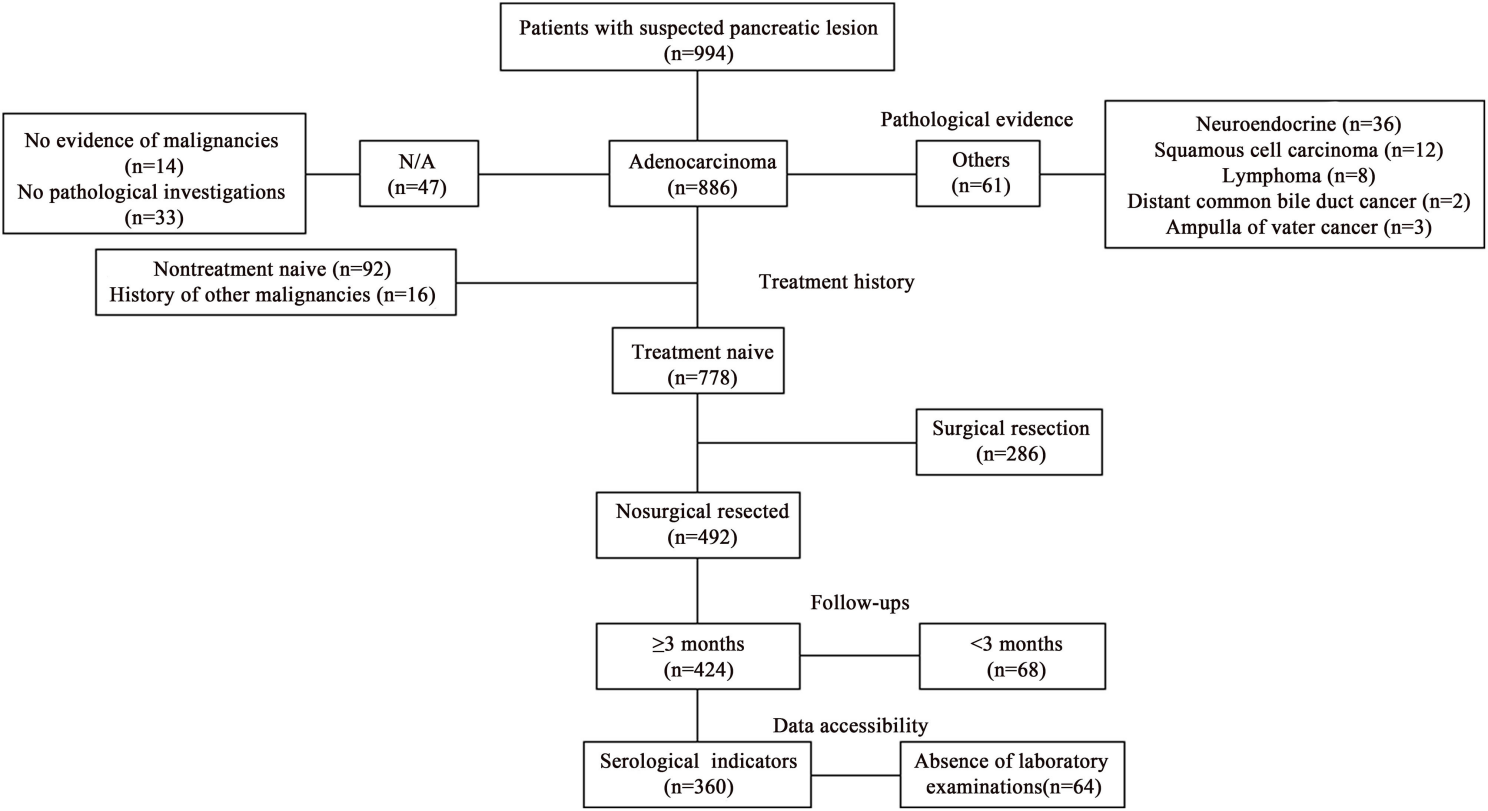
Patient inclusion flow.

All 360 patients were diagnosed and received primary treatment at Fudan University Shanghai Cancer from January 2014 to October 2018 and were included in this retrospective study based on the following criteria: (1) pathologically confirmed pancreatic adenocarcinoma; (2) diagnosed with unresectable diseases, stage III and IV tumor according to the 8th edition of the American Joint Committee on Cancer (Chicago, IL, USA) (15), based on radiological imaging examinations including contrast-enhanced abdominal CT scans, magnetic resonance imaging (MRI) and/or magnetic resonance cholangiopancreatography (MRCP); (3) received gemcitabine-based systemic chemotherapy; (4) no history of other primary malignancies; and (5) follow-up for more than 3 months. The exclusion criteria were: (1) incomplete clinicopathological data; (2) follow-up for < 3 months; and (3) acute inflammatory disease, including diseases that can cause secondary diabetes, such as hepatogenic diabetes, Cushing's syndrome, glucagonoma, pheochromocytoma, hyperthyroidism and somatostatin, and other types of diabetes, high blood sugar caused by drugs, etc. (Figure 1).

### Clinical Variables

Data on patient demographics, tumor location, jaundice and PTCD, and stage were obtained from medical records in the institutional electronic medical record database. Laboratory parameters including neutrophil counts, lymphocyte counts, monocyte counts, platelet counts, fasting blood glucose, CA199, and CEA were collected. All laboratory parameters were assayed during routine workups before cancer diagnostic interventions. The blood glucose-to-lymphocyte ratio (GLR), neutrophil/lymphocyte ratio (NLR), platelet/lymphocyte ratio (PLR), and lymphocyte/monocyte ratio (LMR) were calculated.

### Follow-Ups

The overall survival (OS) was defined as the interval between the date of pathologically confirmed diagnosis and the date of death or the date of the last follow-up. All patients in this study were followed up regularly by an independent researcher with a telephone interview or medical records review. Follow-up was obtained once every 3 months in the first year since the initial diagnosis, then every 6 months. Censoring occurred if patients were still alive upon the last follow-up or died of other causes. Follow-up was terminated on December 20, 2018.

### Statistical Analyses

All statistical analyses were conducted using the Statistical Package Social Science Version 19.0 (SPSS, Inc.). Normally distributed continuous variables are expressed as the mean ± standard deviation (SD) and were analyzed by Student's *t*-test. Non-normally distributed continuous variables are expressed as the median (range) and were analyzed by the Wilcoxon rank sum test. Categorical variables are presented as a frequency (%) and the correlations were determined with the Pearson's chi-square test. The overall survival (OS) was calculated from the date of pathological diagnosis to the date of death or last follow-up. The Kaplan-Meier method was used to compare OS between patients in different groups, and the log-rank test was used to assess the associations of prognostic factors with survival. Univariate and multivariate analyses were performed using the Cox proportional hazards regression model to identify the independent prognostic factors for survival. The hazard ratios (HRs) estimated by the Cox regression model were reported as relative risks with a corresponding 95% confidence interval (CI). A *P* < 0.05 was considered statistically significant. The nomogram plot and C-index analyses fit of the Cox regression model were plotted using the statistical software package R3.6.0.

## Results

### Patient Characteristics and Clinical Features

Among the 360 patients included in the present study, the median age was 61 (ranging from 28 to 84 years), with 221 (61.4%) male patients compared with 139 (38.6%) female patients. According to AJCC staging, 106 (29.4%) patients were at III stage and 254 (70.6%) patients were at IV stage. A total of 143 (39.7%) patients had tumor masses located in the head and neck of the pancreas, and 217 (60.3%) patients had tumor masses located in the body and tail of the pancreas. All patients received gemcitabine-based palliative chemotherapy. Among them, 238 patients in the training group and the remaining 122 patients were in the validation group. The patients' characteristics in the experimental and validation groups are summarized in Table 1. There were no significant differences between the two groups.

**Table 1:** Clinical information for data sets.

**Variables**		**Training cohort**	**Validation cohort**	***P*-value**
		**(*n* = 238,%)**	**(*n* = 122,%)**	
Age (years)	Mean ± SD	60.88 ±9.71	60.87 ± 10.58	0.993
Gender	Male	143 (60.08)	78 (63.93)	0.478
	Female	95 (39.92)	44 (36.07)	
Location	Head	93 (39.08)	50 (40.98)	0.726
	Body, tail	145 (60.92)	72 (59.02)	
TNM stage	III	69 (28.99)	37 (30.33)	0.792
	IV	169 (71.01)	85 (69.67)	
Lymphocyte	Mean ± SD	1.52 ± 0.63	1.49 ± 0.57	0.729
Monocyte	Mean ± SD	0.50 ± 0.28	0.48 ± 0.23	0.693
Neutrophil	Mean ± SD	4.75 ± 2.63	4.82 ± 2.39	0.814
PLT	Mean ± SD	206.20 ± 98.71	202.02 ± 74.60	0.681
GLU	≤6.1 mmol/l	115 (48.32)	64 (52.46)	0.457
	>6.1 mmol/l	123 (51.68)	58 (47.54)	
CA199	0.00–27.00 U/ml	36 (15.13)	23 (18.85)	0.366
	>27.00 U/ml	202 (84.87)	99 (81.15)	
CEA	0.00–5.20 ng/ml	110 (46.22)	64 (52.46)	0.262
	>5.20 ng/ml	128 (53.78)	58 (47.54)	
Jaundice	None	199 (83.61)	104 (85.25)	0.688
	Yes	39 (16.39)	18 (14.75)	
PTCD	None	203 (85.29)	103 (84.43)	0.827
	Yes	35 (14.71)	19 (15.57)	

### Cut-Off Values of the GLR and Other Systemic Inflammatory Response Parameters

ROC curve analysis of the GLR showed that the optimal cut-off value was 4.452, the sensitivity was 66.4%, and the specificity was 77.6% (AUC 0.768; *P* < 0.001). The optimal cut-off value for NLR, PLR and LMR was 3.000 (AUC 0.650; *P* < 0.001), 130.357 (AUC 0.603; *P* = 0.006) and 2.798 (AUC 0.618; *P* = 0.002), respectively, as shown in Table 2.

**Table 2:** ROC curve.

**Variable**	**Cut-off**	**Sensitivity**	**Specificity**	**Area under**	***P*-value**
	**point**			**curve**	
GLR	4.452	0.664	0.776	0.768	<0.001
NLR	3.000	0.611	0.664	0.650	<0.001
PLR	130.357	0.656	0.523	0.603	0.006
LMR	2.798	0.776	0.473	0.618	0.002

### Effect of GLR and Systemic Inflammatory Response Markers on OS

**Figure 2:**
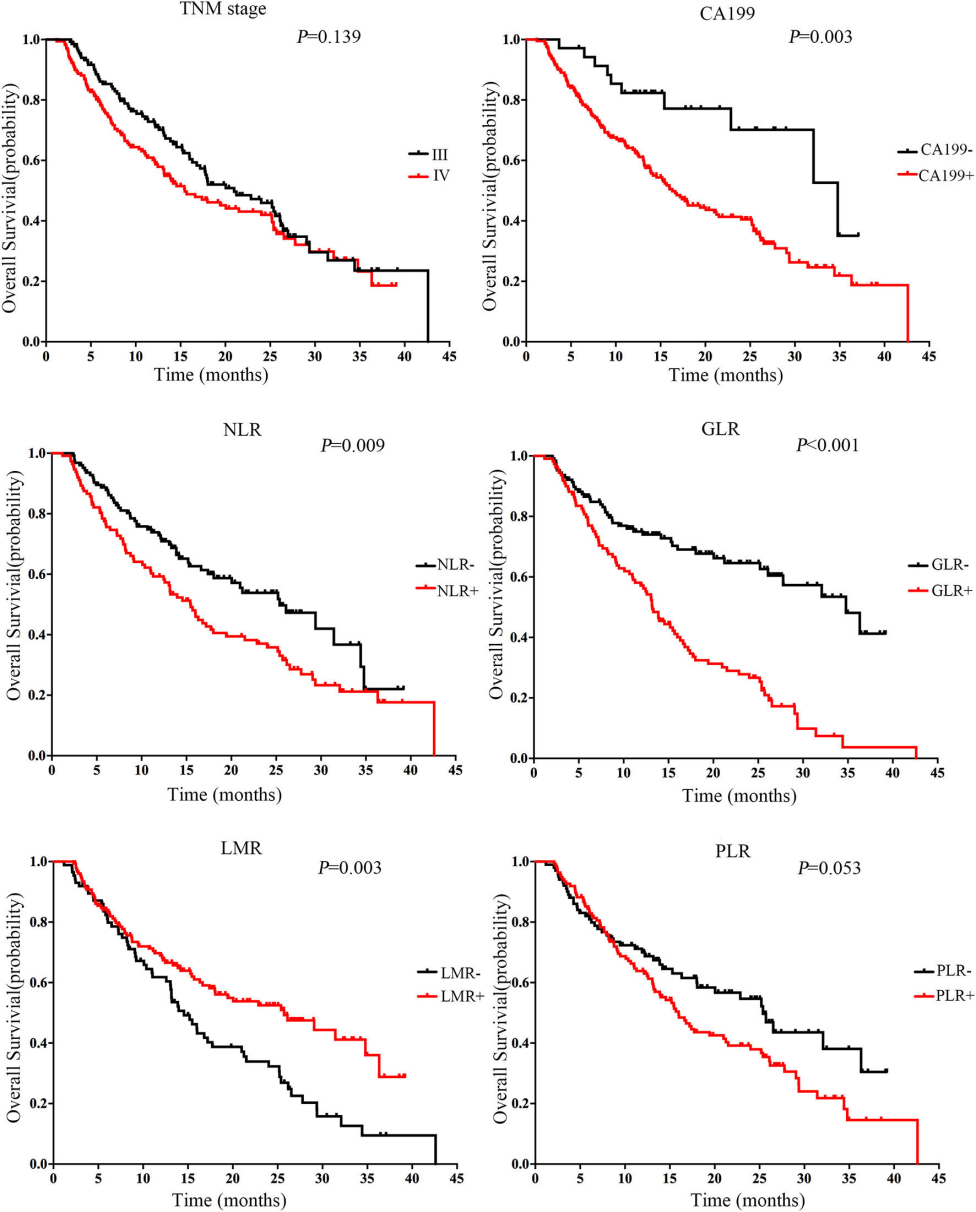
Association between markers and overall survival (OS) in patients with inoperable pancreatic cancer in the training cohort (*N* = 238). Kaplan-Meier curves for OS of all cases in the training cohort. The median level was selected as the cut-off between the low and high levels. The *P*-value was determined using the log-rank test.

For each marker, the cut-off value was used to analyze its association with OS. Survival analysis showed that patients with GLR^+^ (GLR > 4.452) had a shorter survival time than patients with GLR^−^ (GLR ≤ 4.452). The Kaplan-Meier analysis indicated that a higher GLR was associated with a shorter OS (*P* < 0.001, Figure 2). Survival analyses found significant differences among the survival curves of patients with positive CA199, PLR, LMR, and NLR (*P* < 0.001).

### Independent Prognostic Factors of Patients With Inoperable Pancreatic Cancer

Univariate analysis indicated that high CA199, high GLU, high GLR, and high NLR were risk factors for OS in the training cohort (*P* < 0.05). A high LMR was associated with a longer OS (*P* < 0.001). Age, gender, jaundice, PTCD, and tumor location had no prognostic value for OS. Multivariate analysis found that GLR (HR: 2.597, 95% CI: 1.728–3.904, *P* < 0.001) and CA199 (HR: 2.484, 95% CI: 1.295–4.765, *P* < 0.05) were the independent prognostic factors for OS. The factors affecting the survival time of patients with TNM staging were statistically significant (HR: 1.666, 95% CI: 1.103–2.516, *P* < 0.05). The detailed results of the univariate and multivariate analyses are presented in Table 3.

**Table 3:** Cox regression analysis.

**Variable**	**Univariate analysis**		**Multivariate analysis**		
	***β***	***P*****-value**	**HR**	**HR 95% CI**	***P*****-value**
Age	0.005	0.603			
Gender	−0.084	0.638			
Location	0.302	0.098			
Jaundice	−0.096	0.683			
PTCD	−0.446	0.105			
CEA(> 5.20 ng/ml)	0.330	0.064			
PLR(> 130.357)	0.355	0.054			
GLU(>6.1 mmol/l)	0.411	0.022	1.115	0.771–1.614	0.562
TNM stage	0.414	0.037	1.666	1.103–2.516	0.015
CA199(>27 U/ml)	0.941	0.004	2.484	1.295–4.765	0.006
GLR(>4.452)	1.040	<0.001	2.597	1.728–3.904	<0.001
NLR(>3.000)	0.460	0.010	1.010	0.673–1.517	0.961
LMR(>2.798)	−0.523	0.003	0.820	0.554–1.214	0.321

*HR, Hazard Ratio; CI, Confidence Interval*.

### Prognostic Nomogram for Median Survival and OS

**Figure 3:**
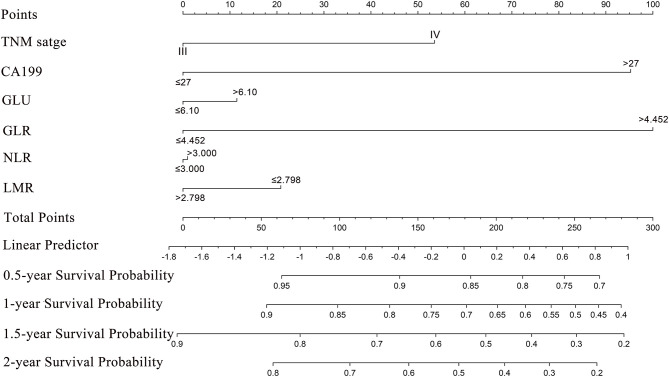
Nomogram for predicting median survival time and survival probability of inoperable pancreatic cancer patients. To use the nomogram, an individual patient's value is located on each variable axis, and a line is drawn upward to determine the number of points received for each variable. The sum of these numbers is located on the Total Points axis, and a line is drawn downward to the survival axes to determine the estimated median survival time and survival probability.

Based on the multivariate analysis, a nomogram that involved all of the independent prognostic factors mentioned above was constructed to predict the OS of the patients. A higher point score indicates a shorter OS. The nomogram showed that GLR contributed the most to prognosis, followed by CA199, TNM stage, etc. (Figure 3). Each of these independent prognostic factors was assigned with a score according to the point scale bar. The sum of the point score projected on the bottom total point scale bar, and the respective location on the median survival bar and the survival bar indicates the median OS and survival probability. Scoring according to the preoperative indicators of the patients can assess the prognosis of the patients and give reasonable suggestions.

### Validation of the Predictive Accuracy of the Nomograms

**Figure 4:**
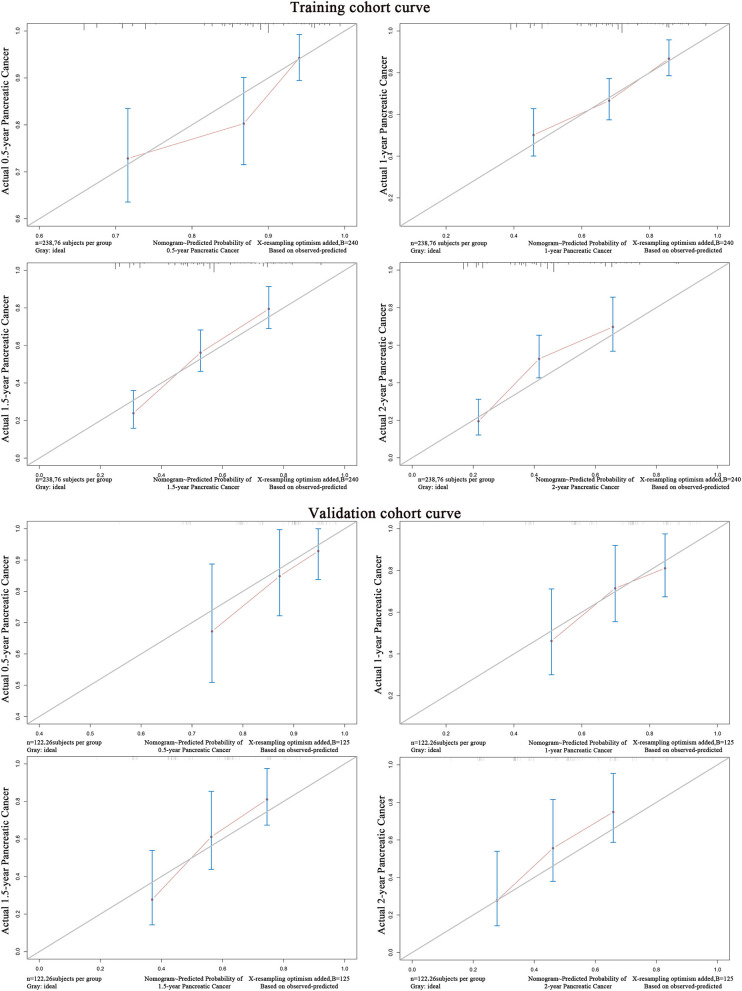
The calibration curve for predicting survival probability of inoperable pancreatic cancer patients in the training cohort and validation cohort.

In the validation cohorts, the predictive accuracy of the model was evaluated based on the COX regression model of the training group by the C-index fitting model. The C-index of the training cohort and validation cohort were 0.674 and 0.671 (Table 4). The model was fitted according to the actual survival state of the patients. The difference was statistically significant (*P* < 0.001). The ROC curves of the training cohort and the verification cohort showed a good relationship, suggesting the nomogram predictions and the actual observations for the probability of median survival and 1-year survival were very similar. The calibration curves for predicting survival probability in the training cohort and validation cohort are shown in Figure 4. Both of the curves showed good agreement between the nomogram prediction and the actual observations for 1-year and 1.5-year OS.

**Table 4:** Regression model fitting verification.

**Variable**	**Training cohort**	**Validation cohort**
	**(*n* = 238,%)**	**(*n* = 122,%)**
C-Index	0.674	0.671
SD	0.049	0.089
Dxy	0.339	0.339
Z	6.94	3.79
*P*-value	<0.001	<0.001

### Risk Stratification of OS

**Figure 5:**
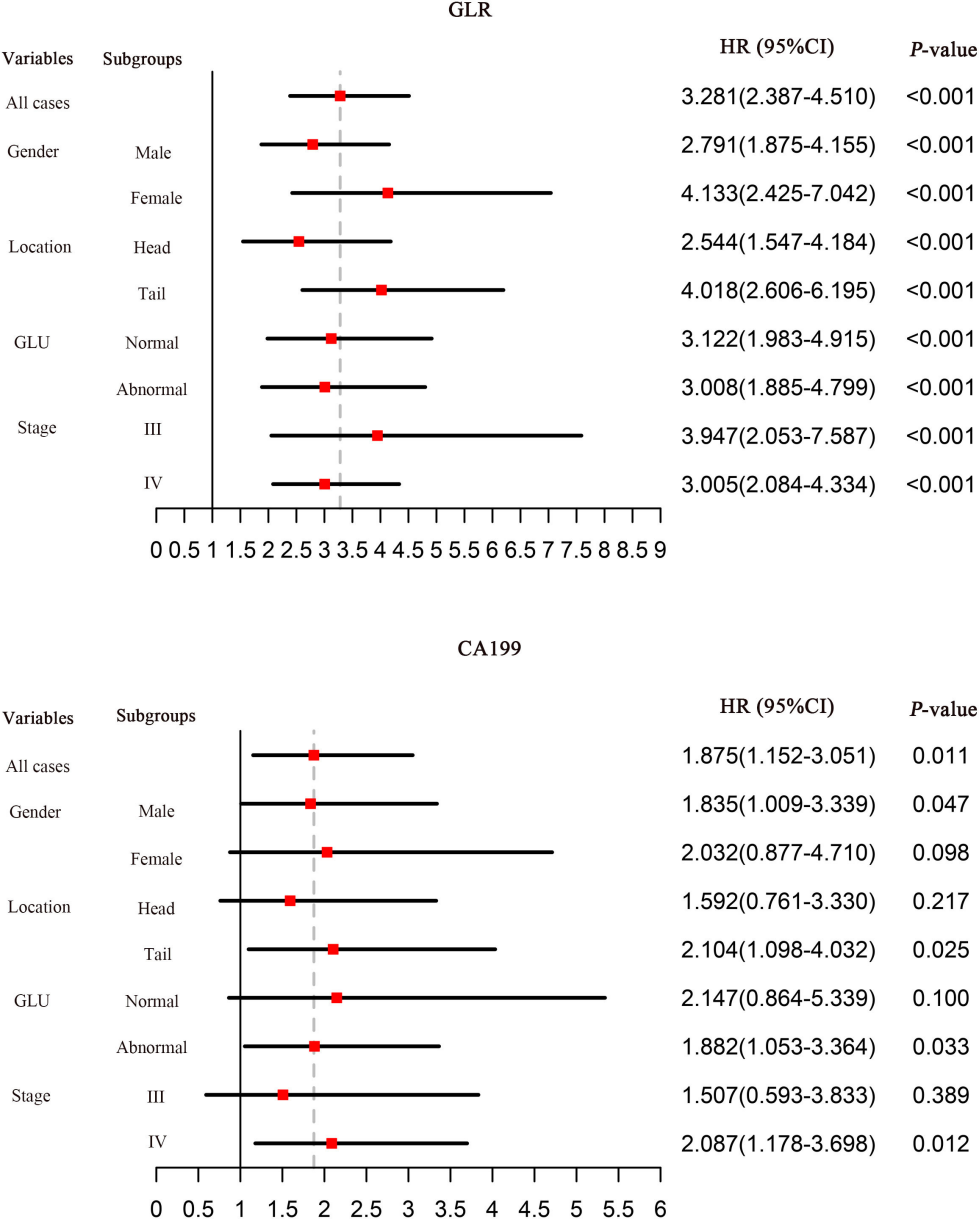
Hazard ratios (HRs) of prognostic markers for OS in different patient subgroups in the training cohort. HRs were calculated by comparing patients with low values to those with high values. HRs with a 1.0 line indicate a meaningless outcome.

The cut-off values of the total points determined by the nomogram and Cox regression analysis showed that GLR and CA199 are independent risk factors for OS in patients with pancreatic cancer. The predictive values of GLR and CA199 are independent of all other clinical variables tested, while the predictive values of NLR and LMR are affected by the other variables and are not independent risk factors. GLR can predict patient survival in subgroups of untreated pancreatic cancer patients and is superior to CA199 (Figure 5).

## Discussion

In the present study, we established a correlation between GLR and systemic inflammatory response markers with prognosis in patients with inoperable pancreatic cancer. The results showed that elevated GLR and PLR were significantly associated with a shorter OS. We further established a nomogram model incorporating various hematological factors and clinical characteristics to provide a more accurate prediction that could delineate the large differences in survival among individuals with inoperable pancreatic cancer. Our model showed superior predictive accuracy to the existing TNM staging system. GLR can be used as an independent prognostic factor for pretreatment patients and may provide valuable information for clinical decision making and tailoring the best treatment strategy for individualized treatment.

Systemic inflammation has been associated with the initiation, development, and progression of many cancers. In pancreatic cancer, the elevation of NLR and PLR are independent prognostic factors predicting a poor outcome (16–18), and LMR predicts a better outcome (19, 20). Consistently, these prognostic factors were significantly associated with overall survival in the univariate analysis in the present study. However, after the exclusion of other variables, CA199, CEA, NLR and LMR were not independent factors associated with the overall survival of patients with inoperable pancreatic cancer. To our knowledge, this study is the first to prove that GLR is a prominent factor associated with the survival of patients with inoperable pancreatic cancer.

This study demonstrates the prognostic significance of GLR in inoperable pancreatic cancer. Altered glucose metabolism is a hallmark of cancer. Diabetes is considered a risk factor for pancreatic cancer since nearly one-third of patients with pancreatic cancer have a history of diabetes, and diabetic patients have a higher probability of developing pancreatic cancer (21–23). Furthermore, there is also a correlation between hyperglycemia and poor survival in patients with pancreatic cancer, especially among those with type 2 diabetes (24, 25).

Iarrobino et al. found that pretreatment hyperglycemia in patients with locally advanced pancreatic cancer is associated with a poor prognosis. This study assessed the predictive value of glycemic status and antidiabetic medications for locally advanced pancreatic cancer (26). Another study found that hyperglycemia accelerates pancreatic cancer progression by increasing reactive oxygen species (ROS) levels, promoting epithelial-to-mesenchymal transition (EMT), aggravating hypoxia, and promoting the malignant behavior of pancreatic cancer (27, 28).

The tumor microenvironment is closely related to the occurrence and development of cancer and immune status (29–31). T cell metabolism plays an important role in immune regulation and it plays a key role in anti-tumor immunity (32, 33). In addition, regulatory T cells are favorable under low glucose conditions and can inhibit anti-tumor immune responses (34, 35). Tumor infiltrating lymphocyte levels and the expression levels of immune system genes affect the prognosis of pancreatic ductal adenocarcinoma (36). Conversely, lymphocytes are known to play important roles in suppressing cancer progression by inducing cell death and inhibiting cell proliferation (37, 38).

A glucose-restricted tumor microenvironment induces metabolically adapted, oxidative neutrophils to maintain local immune suppression (39). Consistent with previous findings, peripheral blood neutrophils from cancer patients also display increased immaturity, mitochondrial content, and oxidative phosphorylation. Therefore, it is of value to take into account the synergistic effect of hyperglycemia and immunosuppression on cancer. It is worth noting that the elevated GLR in our study is one of the independent factors affecting the overall survival rate of patients with inoperable pancreatic cancer. GLR also reflects the invasiveness and immune status of the tumor. In the meantime, the nomogram can be used to provide a more scientific assessment of survival risk, providing a basis for individualized treatment and further clinical applications.

This study is based on a retrospective design and therefore has inevitable limitations. First, this is a mono-institutional study with a small sample size, and it only included patients with inoperable pancreatic cancer. Second, tumor staging is included as a dichotomous variable in the nomogram rather than using the subdivisions of TNM staging, which may result in the reduced accuracy of the prediction. The choice of treatment for patients is based on first-line treatment with gemcitabine, but the optimal chemotherapy regimen for patients with pancreatic cancer varies from individual to individual and requires a larger prospective or multicenter study to validate our findings.

In conclusion, pretreatment GLR can be used as an independent prognostic factor for survival in patients with inoperable pancreatic cancer. The proposed nomogram can predict the overall survival rate of patients with inoperable pancreatic cancer with good sensitivity and specificity. Our nomogram provides a helpful tool for clinicians to plan treatment strategies, facilitate individualized treatment, and choose disease management approaches in addition to the traditional indicators and staging systems.

## Data Availability Statement

The datasets generated for this study are available on request to the corresponding author.

## Ethics Statement

The study was approved by the Ethics Committee of Fudan University Shanghai Cancer Center. Written informed of consent was obtained from each participant in accordance with the institutional guidelines.

## Author Contributions

AZ and C-sC mainly responsible for research and design of study. JK was responsible for statistical analysis of data. All authors contributed to the article and approved the submitted version.

## Conflict of Interest

The authors declare that the research was conducted in the absence of any commercial or financial relationships that could be construed as a potential conflict of interest.
